# Cardiac-Autonomic Imbalance and Baroreflex Dysfunction in the Renovascular Angiotensin-Dependent Hypertensive Mouse

**DOI:** 10.1155/2012/968123

**Published:** 2012-11-05

**Authors:** Bianca P. Campagnaro, Agata L. Gava, Silvana S. Meyrelles, Elisardo C. Vasquez

**Affiliations:** ^1^Department of Physiological Sciences, Health Sciences Center, Federal University of Espirito Santo, 29075-910 Vitoria, ES, Brazil; ^2^Biotechnology Graduate Program, Health Sciences Center, Federal University of Espirito Santo, 29075-910 Vitoria, ES, Brazil; ^3^Department of Physiological Sciences, Emescam College of Health Sciences, 29045-402 Vitoria, ES, Brazil; ^4^Lab Transgenes and Cardiovascular Control, Department of Physiological Sciences, Health Sciences Center, Federal University of Espirito Santo, Avenida Marechal Campos 1468, 29042-755 Vitoria, ES, Brazil

## Abstract

Mouse models provide powerful tools for studying the mechanisms underlying the dysfunction of the autonomic reflex control of cardiovascular function and those involved in cardiovascular diseases. The established murine model of two-kidney, one-clip (2K1C) angiotensin II-dependent hypertension represents a useful tool for studying the neural control of cardiovascular function. In this paper, we discuss the main contributions from our laboratory and others regarding cardiac-autonomic imbalance and baroreflex dysfunction. We show recent data from the angiotensin-dependent hypertensive mouse demonstrating DNA damage and oxidative stress using the comet assay and flow cytometry, respectively. Finally, we highlight the relationships between angiotensin and peripheral and central nervous system areas of cardiovascular control and oxidative stress in the 2K1C hypertensive mouse.

## 1. Introduction

The sympathetic nervous system has an excitatory action on the heart and blood vessels, whereas the parasympathetic cardiovagal innervation has an inhibitory action on the heart [1]. Cardiac output and vascular resistance are the main determinants of arterial blood pressure (BP), which is maintained with minimal oscillations by baroreceptors located at the carotid sinus and aortic arch that transmit their signals to integrative medullary areas [1, 2]. Thus, the balanced activity of the efferent autonomic nervous system and arterial baroreceptors is essential for the control of the cardiovascular system to achieve optimal blood flow to the organs of the body.

 As recently reviewed [3], conditions of exaggerated and sustained sympathetic activity, reduced parasympathetic activity, and baroreflex dysfunction are important cardiovascular risks. Over the past decades, our laboratory has shown that these pathological conditions are present as a result of the hypertension induced by the activation of the renin-angiotensin system (RAS) in the rat [4, 5], which are also observed in the RAS-dependent hypertensive mouse [6, 7].

In this paper, we will highlight the characteristics of the murine model of RAS-dependent hypertension, provide new insights into the role played by oxidative oxygen species (ROS) in the integrative brain areas, and discuss which findings are expected to be revealed next. 

## 2. Induction of 2K1C Hypertension in the ****Mouse

 For decades, the rat has been used to study the relationship between RAS and the autonomic nervous system. However, genetic discoveries and advances in molecular biotechnologies have provided the opportunity to develop many mouse models for human diseases. Although a major disadvantage of this animal is the small size, advances in surgical techniques have overcome this limitation, allowing for studies of cerebral [8, 9], cardiac [10], vascular [11], and renal [12] functions. 

**Figure 1:**
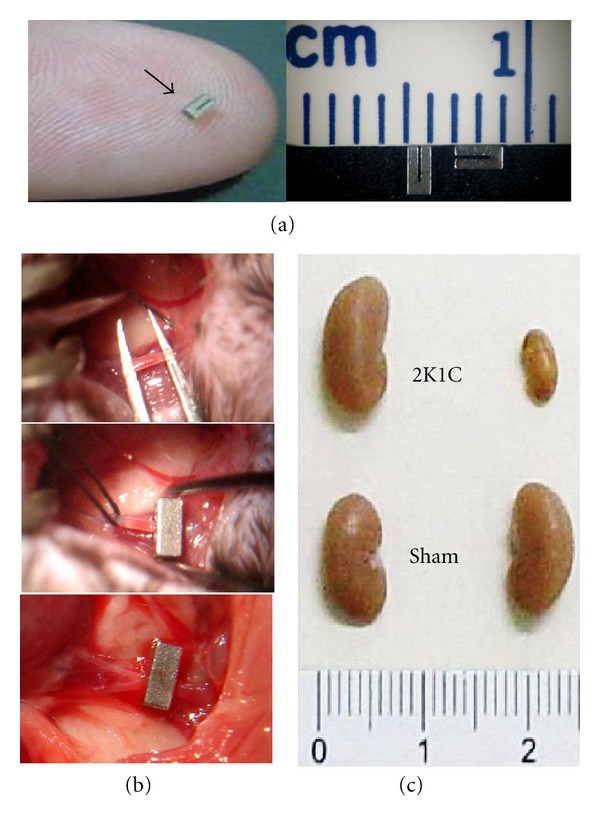
The procedure used to induce angiotensin-dependent hypertension in the mouse. A solid, stainless steel clip with an opening width of 0.12 mm (a) is placed around the left renal artery to cause stenosis (b), which results in the atrophy of the clipped kidney and hypertrophy of the contralateral, nonclipped kidney (c) and hypertension.

 In our laboratory, we used the procedure established by Wiesel et al. [13] to develop a murine model of two-kidney, one-clip (2K1C) hypertension [6, 7, 11, 14]. To minimize variability, a solid stainless steel clip with an opening width of 0.12 mm is placed around the left renal artery (Figure 1) to constrict it and to chronically reduce the perfusion of the left kidney while leaving the other kidney untouched. A mouse body weight of 23 g and clip lumen size of 0.12 mm allows for the induction of hypertension without causing renal infarction [13]. As illustrated in Figure 1, two weeks after clipping, 2K1C mice show atrophy of the clipped (left) kidney and hypertrophy of the contralateral, nonclipped (right) kidney.

Two weeks after renal artery clipping, 2K1C mice already exhibit arterial hypertension (Table 1) with similar levels observed at four weeks [13]. Similar to the 2K1C hypertensive rat that develops cardiac hypertrophy [4, 5], our laboratory has shown a similar phenotype in the 2K1C hypertensive mouse [6, 14]. The development of cardiac hypertrophy is thought to be the result of increased angiotensin II levels through the stimulation of protein and DNA synthesis in cardiac cells [16].

## 3. Systemic and Central Renin-Angiotensin ****Systems

An advantage to using the C57BL/6 mouse for the induction of RAS-dependent hypertension is that it is a prototype of strains with a single renin gene [17], that is, this mouse does not behave differently from the rat in the 2K1C model of renovascular hypertension. As shown in Tables 1 and 2, the high BP in this model is due to a rapid increase in plasma renin levels (~3-fold) in response to a reduction in the perfusion pressure in the stenotic kidney, which secretes renin from juxtaglomerular cells. This is followed by a subsequent increase in plasma angiotensin I, which is further converted to the vasoactive angiotensin II (~4.5-fold). Pressure diuresis and hypertrophy of the contralateral kidney (Figure 1, Table 1) prevents hypervolemia [13, 14]. As recently demonstrated by our laboratory, 2K1C mice also show augmented levels of angiotensin 1-7 (Table 2), which is an angiotensin I metabolite formed by a pathway that is independent of angiotensin-converting enzyme (ACE) [18]. Interestingly, knocking-out the angiotensin 1-7 receptor Mas exacerbates the course of 2K1C hypertension in mice [19]. The observed increase in the level of this peptide in the 2K1C mouse seems to serve as an important endogenous, physiological counterbalancing mechanism that partially attenuates the hypertensinogenic actions of activated RAS [18].

In some brain areas, including the rostral ventrolateral medulla (RVLM), hypothalamic paraventricular nucleus (PVN), and subfornical organ (SFO), a local RAS has been identified to act as a critical mediator of chronic hypertension in the 2K1C mouse model [15, 20, 21]. The SFO is a circumventricular region that has a fenestrated vasculature that could permit the entry of increased circulating levels of angiotensin II in addition to residual locally produced angiotensin, leading to the stimulation of the local production of angiotensin II in other brain areas protected by the blood-brain barrier [20]. 

## 4. Imbalance of the Cardiac Autonomic Nervous System 

An imbalance of the autonomic nervous system, as often occurs in conjunction with several cardiovascular diseases, affects BP and HR variability [22, 23], which may be associated with targeted organ damage and an increased risk of morbidity and mortality [3]. Central areas that are involved in the autonomic control of the cardiovascular system include the rostral ventrolateral and ventromedial medulla (RVLM and RVMM), the caudal ventrolateral medulla (CVLM), PVN, and SFO [24–26]. The signals that are generated in the sinoaortic baroreceptor endings are transmitted through the afferents of cranial nerves XI and X to the nucleus tractus solitarius (NTS), followed by the CVLM, and are processed in the RVLM. The RVLM also integrates inputs from the SFO and PVN, providing a major input to the preganglionic neurons of the sympathetic nervous system [15, 20, 27]. Thus, through the integrative processing of central areas, the autonomic sympathetic and parasympathetic nervous systems provide control to the cardiovascular system and the optimal perfusion of organs in accordance with their metabolic needs. 

**Figure 2:**
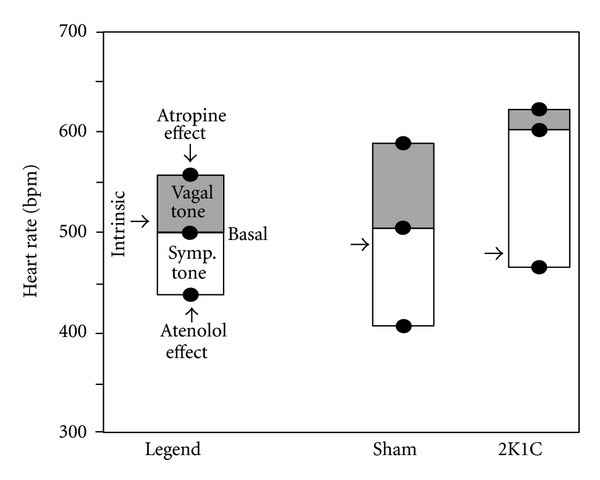
Typical imbalance of cardiac autonomic tones in the angiotensin-dependent hypertensive mouse. The cardiovagal tone is represented by the tachycardia observed following the administration of the muscarinic blocker atropine, and the cardiac sympathetic tone is represented by the bradycardia observed after administering the *β*-adrenergic blocker atenolol. The heart rate after the double blockade indicates the intrinsic heart rate.

The parasympathetic cardiovagal and sympathetic tones in the mouse have traditionally been assessed through pharmacological methods involving a *β*_1_-blocker (atenolol), a muscarinic, cholinergic receptor blocker (atropine methyl nitrate) or a double blockade of those receptors [28, 29]. The increase in HR after administering atropine reflects the cardiovagal tone present under baseline resting conditions, and the decrease in HR after atenolol administration reflects cardiac sympathetic tone (Figure 2); a double blockade enables the determination of the intrinsic HR. In the wild-type mouse under resting conditions, a balance between the sympathetic and parasympathetic activities has been reported [7], with a predominance of the sympathetic tone over the cardiovagal tone under special conditions [30].

As shown in Figure 2, the autonomic control of HR in 2K1C hypertensive mice is characterized by an increased cardiac sympathetic tone, whereas the parasympathetic cardiovagal tone is decreased when compared to sham mice [7]. This condition in humans and animal models of cardiovascular diseases represents a major risk factor for cardiovascular mortality [3]. Angiotensin II mediates the increased activity of the sympathetic nerve to the heart in experimental models of RAS-dependent hypertension [32]. In rats, it has been suggested that an infusion of angiotensin II contributes to tachycardia by increasing the intrinsic HR [33]. However, this does not appear to be the case in the 2K1C mouse model, which shows tachycardia without marked changes in this hemodynamic parameter [6]. Considering that the neuronal nitric oxide synthase- (nNOS-) deficient mouse exhibits tachycardia primarily due to abnormal cardiac autonomic control [34] and that endothelial nitric oxide synthase (eNOS) gene therapy restores the basal HR in 2K1C mice [7], it is possible that nitric oxide (NO) plays a role in the autonomic control of HR in this model of RAS-dependent hypertension.

## 5. Baroreflex Dysfunction

**Figure 3:**
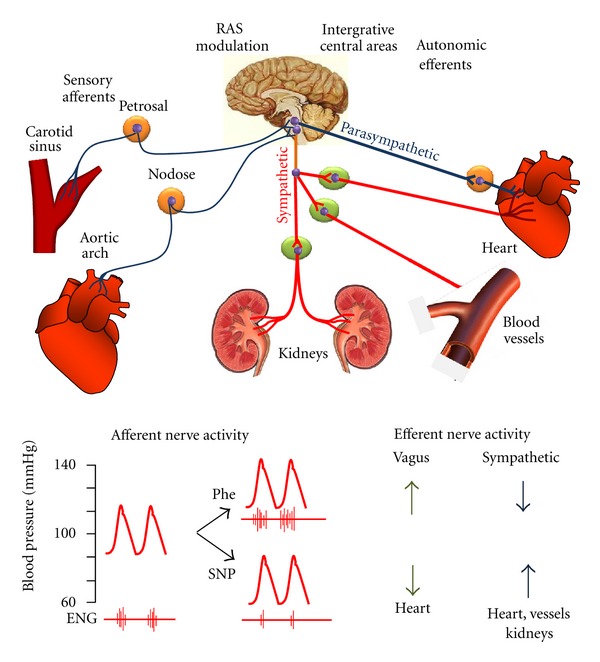
Neuroreflex control of circulation. The top panel illustrates the main neural components of the baroreflex arch. The bottom panel shows a schematic illustration of evaluation of the baroreflex function using the vasoactive agent phenylephrine (Phe) and sodium nitroprusside (SNP) in the murine model of renovascular hypertension. ENG, electroneurogram. The scheme is based on previous publications [3, 31].

Among the neural systems that control cardiovascular function, the baroreflex is a neural mechanism that acts moment-to-moment to maintain BP with minimal fluctuations [1]. With each arterial systole, mechanosensitive nerve endings located at the carotid sinuses and the aortic arch generate bursts of action potentials that are transmitted to the NTS in the medulla oblongata. Here, the signals are integrated and result in the maintenance of a balanced parasympathetic outflow to the heart and a sympathetic outflow to the heart, vessels and kidneys (Figure 3, top panel). As illustrated in Figure 3 (bottom panel), an immediate rise in BP evokes a reflexive increase in cardiovagal inhibitory activity and a decrease in cardiac and vascular sympathetic excitatory activity, resulting in an immediate correction of BP. Conversely, in response to a rapid decrease in BP, cardiovagal activity is diminished and cardiac and vascular sympathetic activity increase to return the BP to normal values.

In our laboratory, the sensitivity of the baroreflex has been traditionally assessed through pharmacological approaches in conscious animals. An acute, phenylephrine- (Phe-) induced increase in BP leads to an increase in the number of action potentials generated at each discharge and, consequently, to a reflexive increase in parasympathetic and a decrease in sympathetic nerve activities. The opposite is observed during an acute, sodium nitroprusside- (SNP-) induced decrease in BP. Peak values of mean arterial pressure (MAP) and HR in response to Phe and SNP injections are fitted to a sigmoidal logistic equation, which is used to determine the gain (first derivative of the curve) and the maximum reflex tachycardia (upper plateau) and reflex bradycardia (lower plateau) [5, 35]. Considering the small size of the mouse, it is more appropriate to evaluate the baroreflex function in conscious mice by injecting a single dose or by slowly infusing Phe and SNP to avoid volume overloading. 

**Figure 4:**
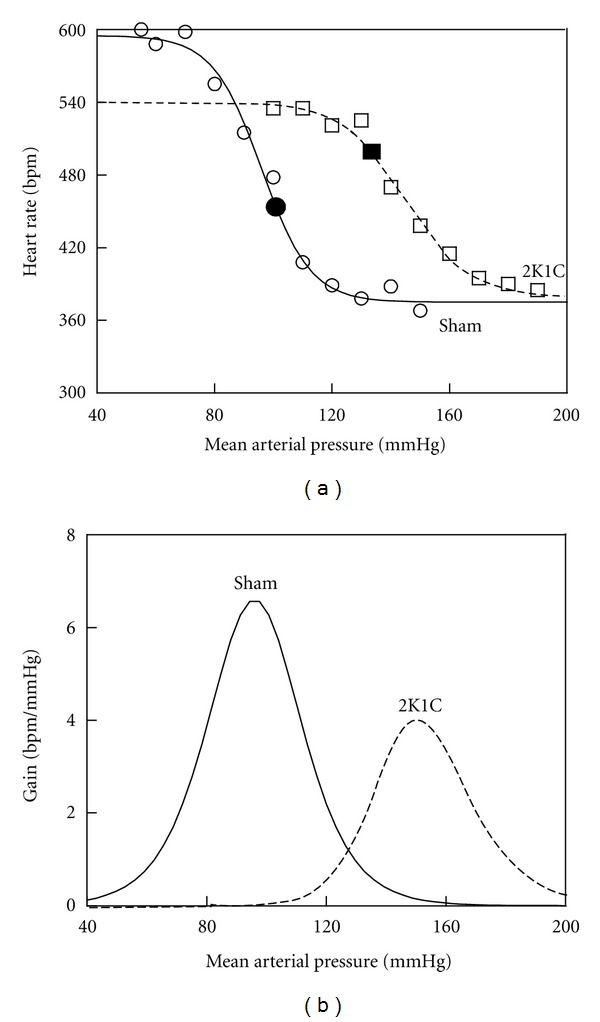
Plots showing typical reflex heart rate changes as a function of drug-induced changes in arterial pressure using logistic, sigmoidal-fitting barocurve analysis (a) and baroreflex gains calculated from the first derivative of the sigmoid function (b) comparing 2K1C with sham mice. The small circles and squares indicate individual changes in heart rate in response to every 10 mmHg of drug-induced changes in arterial pressure.

 A disruption in the balance between parasympathetic and sympathetic tones, as discussed above, can lead to an impairment in baroreflex sensitivity, as has been demonstrated by our laboratory in different models of hypertension [5, 36–38].  Figure 4 shows representative sigmoidal barocurves of a 2K1C mouse compared to a sham animal. The 2K1C mouse curve is shifted to the right of the sham mouse, closely following the high levels of MAP at the midpoint of the curve. The lower slope of the fitting curve indicates impaired baroreflex sensitivity in the 2K1C mouse. We exclude the possibility that the decreased baroreflex sensitivity in 2K1C mice could be due to a limited chronotropic reserve to respond to increases in HR, as the upper plateau of the barocurve of 2K1C mice was below of that observed for sham mice. Based on observations from our laboratory and others, a reasonable explanation for this finding is that, apart from its pressure effect, adventitial angiotensin II and its AT_1_ receptors at the aortic arch (and probably at the carotid sinus) act by decreasing the sensitivity of aortic afferents during physiological changes in BP, thus contributing to the impairment of the baroreflex function in cardiovascular diseases [31, 39, 40]. Interestingly, in the rat, central endogenous angiotensin 1-7 has been shown to counterbalance the angiotensin II-induced baroreflex dysfunction [41]. Moreover, a lack of the angiotensin 1-7 Mas receptor-induced baroreflex dysfunction in mice [42]; however, this has not yet been investigated in the renovascular 2K1C mouse model. 

## 6. DNA Damage and Oxidative Stress

There is mounting evidence that increased oxidative stress contributes to increased cardiac and vascular sympathetic tone and decreased baroreflex sensitivity in cardiovascular diseases, including hypertension, as recently reviewed elsewhere [3, 21]. Because ROS play a crucial role in RAS signaling [21, 43, 44], a key mechanism by which angiotensin II influences the heart and vessel function could be through its ability to activate ROS production [20, 45]. ROS have been shown to mediate the actions of angiotensin II at the ganglionic [46] and central nervous system levels, resulting in excessive sympathetic drive to the heart [45, 47, 48]. In our laboratory, we currently use the comet assay associated with dihydroethidium (DHE) staining to evaluate oxidative stress in different cells and tissues of the 2K1C hypertensive mouse.

The intracellular oxidation of DHE to the fluorescent dye ethidium has been previously used as an indicator of superoxide generation [49]. DHE is freely permeable to cell membranes and can be directly oxidized to ethidium bromide in cell cytoplasm by the superoxide anion [50, 51]. Ethidium bromide becomes trapped in the nucleus by intercalating within DNA, leading to an increase in ethidium fluorescence in the cell nucleus. DHE itself fluoresces blue in the cell cytoplasm, while the oxidized form ethidium fluoresces red following DNA intercalation. Blood cells can be used to assess ROS generation by superoxide detection with DHE.

The most important, biologically active oxidant in the cardiovascular system, superoxide is a highly reactive and short-lived radical responsible for ROS generation. In addition, it can interact with nearby molecules such as DNA, and thus play a key role in inducing DNA oxidative damage [52, 53]. The comet assay is recognized as a versatile and sensitive method for quantifying and analyzing DNA fragmentation in individual cells and can be used to assess DNA exhibiting oxidative damage. The basic principle of the comet assay is the migration of DNA in an agarose matrix under electrophoretic conditions. As a result of this migration, the cells look like comets under microscopic visualization, with a head (intact DNA) and a tail containing DNA fragments. Individual blood cells are embedded in low-melting-point agarose and spread on a common microscope slide. Membranes, soluble cell constituents, and histones are removed by lysing with detergent and high-salt solution. Following the lysis procedure, the slides are placed in an electrophoresis chamber filled with an alkaline buffer (pH > 13) for DNA unwinding. Then, the DNA undergoes electrophoresis, allowing for the migration of DNA fragments out of the nucleus in an electrical field towards the anode. Staining is usually performed with a DNA-specific fluorescent dye such as ethidium bromide and observed using a fluorescence microscope. The result of this migration is a bright fluorescent head and tail that gives the appearance of a comet. The relative content of DNA in the tail indicates the amount of DNA damage. 

**Figure 5:**
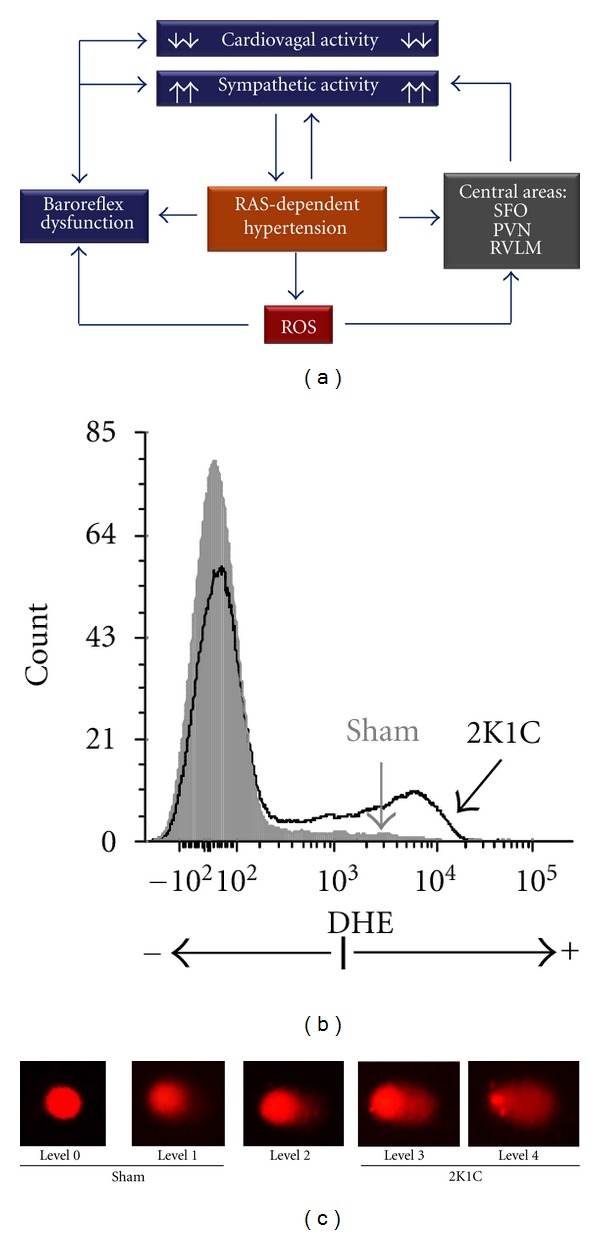
Relationship between RAS activation, ROS production, and baroreflex dysfunction in the 2K1C mouse. (a) Effects of renal clipping-induced high plasma levels of angiotensin II on peripheral and central neural areas controlling cardiovascular function mediated by reactive oxygen species (ROS); (b) typical flow cytometric analysis with the dihydroethidium assay (DHE) showing elevated production of superoxide in 2K1C mice; (c) comet assay illustrating the detection of greater levels of DNA damage (comet-tail fragmentation) in the 2K1C mice.

As illustrated in Figure 5, the incidence of genomic fragmentation is visually scored into five levels according to the comet-tail size. RAS-dependent hypertensive mice predominantly present comets with elevated DNA damage levels (3 and 4) in whole blood cells. In addition, flow cytometric analysis of blood cells shows an augmentation of DHE staining in these animals, which indicates that 2K1C hypertension increases superoxide generation, in turn, leading to DNA fragmentation in whole blood cells. Ongoing studies are focused on the effects of RAS-induced hypertension in the cells of different tissues.

## 7. The Relationship between RAS, ROS, and the Autonomic Control of Cardiovascular ****Function

Our finding of increased DNA damage and ROS production in the 2K1C mouse is in agreement with other studies that found an accumulation of superoxide at the ganglionic level [46] and in different brain integrative areas such as the PVN in this murine model of RAS-induced hypertension [15, 20, 45]. It is thought that the involvement of the PVN in 2K1C hypertension occurs through the activation of RVLM-projecting parvocellular neurons in this region, leading to increased sympathoexcitation [21, 45]. Based on the above data, a plausible mechanism involved in baroreflex dysfunction and the imbalance of the parasympathetic (diminished) and sympathetic (increased) tones appears to be an excessive generation of ROS in the circulatory system and in both the peripheral and central components of the baroreflex. As recently reviewed, ROS is an insidious and ubiquitous promoter of sympathoexcitation and baroreflex dysfunction that can accelerate or worsen cardiovascular disease processes and cardiovascular risks [3, 21].

## 8. New Insights in Therapeutic Approaches to ****Improve Cardiovascular Function in 2K1C ****Mice

Although therapies have been aimed at nullifying the undesirable effects of angiotensin II, some investigators have focused on demonstrating the importance of the counterbalanced effects of angiotensin 1-7 [18], which is increased in the 2K1C hypertensive mouse [14]. For example, it has been shown that enalapril treatment increases the sensitivity of the baroreflex in the rat, and that this effect was reversed by an i.c.v. infusion of the selective angiotensin 1-7 antagonist, D-Ala7-Ang-1-7 (A-779) [41]. Similar results were observed when A-799 was injected into the CVLM in 2K1C hypertensive rats [54]. Recently, others have shown that the knockout of the angiotensin 1-7 Mas receptor in mice exacerbates the course of 2K1C hypertension [19]. Considering the identification of the angiotensin-converting enzyme homologue ACE2 as an angiotensin peptide-processing enzyme and of Mas as a receptor for angiotensin 1-7, this axis is a putative target for the development of new cardiovascular drugs [18]. Furthermore, there has been a lack of studies evaluating the effects of peripheral and central manipulation of angiotensin 1-7 on cardiac autonomic tones and the baroreflex function in the 2K1C mouse model. 

As recently reviewed [21], there is growing evidence that acute or chronic antioxidant treatment decreases BP and sympathetic activity and improves the baroreflex control of HR in 2K1C rats. Furthermore, tempol or vitamin C administered systemically or into the RVLM or PVN diminishes BP and sympathetic activity [21], highlighting the pivotal role played by central integrative areas controlling cardiovascular function in RAS-dependent renovascular hypertension. 

Some gene therapies have also been tested in studies of 2K1C hypertensive mice, but up until now, no favorable results have been observed. For example, Gava et al. [7] used gene therapy in 2K1C mice and observed that it prevented the development of hypertension but not baroreflex dysfunction. Burmeister et al. [15] tested the hypothesis that excessive superoxide anion production in the PVN contributes to the development and maintenance of renovascular hypertension by delivering an adenovirus encoding superoxide dismutase (AdCuZnSOD) to the PVN. They observed that this prevents the elevation in superoxide anions and abolishes renovascular hypertension. However, this approach has not yet been used to evaluate effects on baroreflex dysfunction in the 2K1C mouse. 

## 9. Conclusions and Perspectives

In the past few years, the mouse model of 2K1C hypertension has greatly contributed to the understanding of the relationships between RAS and neural control of cardiovascular function. In addition to the actions of systemic angiotensin II, it has been demonstrated thata local RAS in RVLM, PVN and SFO brain areas act as a critical mediator of chronic hypertension in this experimental model. The 2K1C hypertensive mouse exhibits a cardiac autonomic imbalance characterized by an increased sympathetic tone and a decreased vagal tone, beyond impaired baroreflex sensitivity. In addition to the demonstrations that ROS play a crucial role in RAS signaling at the ganglionic and central nervous system levels, there are growing evidences that DNA damage and increased oxidative stress contribute to the increased cardiac and vascular sympathetic tone and decreased baroreflex sensitivity in the renovascular hypertension. It is well known that angiotensin II increases superoxide production through the activation of NADPH oxidase. Gene therapies by delivering an adenovirus encoding eNOS or enzymes that prevent the elevation of superoxide anions have shown to improve the cardiac autonomic control of HR and baroreflex sensitivity and to prevent renovascular hypertension in the murine model. Although therapies have been aimed at nullifying the undesirable effects of angiotensin II, a putative target for the development of new cardiovascular drugs is the angiotensin 1-7 which induces the release of NO and diminishes NADPH oxidase activation, counteracting the effects of angiotensin II. Therefore, future studies should address potential strategies to decrease oxidative stress and to prevent or restore the cardiac autonomic balance and the baroreflex function in the mouse model of renovascular hypertension.

## Figures and Tables

**Table 1:** Cardiovascular parameters in 2K1C mice compared to Sham mice, two weeks after clipping.

Parameter	Sham	2K1C	Reference no.
Mean arterial pressure (mmHg)	~100–115	~120–135	[13, 14]
Heart rate (bpm)	~500–570	~650	[14]
Cardiac weight/body weight index (mg/g) wet (dry)	~4.1 (~1.0)	~4.5 (~1.3)	[13, 14]
Nonclipped kidney weight (mg)	140–160	170–210	[14, 15]
Clipped kidney weight (mg)	150–160	70–90	[14, 15]

**Table 2:** Average values of plasma renin, angiotensin I, II and 1-7 in 2K1C mice compared to Sham mice, two weeks after clipping.

Parameter	Sham	2K1C	Reference no.
Renin (ng Ang I/mL/hr)*	~1000	~3000	[13]
Angiotensin I (pmol/mL)	~80	~160	[14]
Angiotensin II (pmol/mL)	~30	~140	[14]
Angiotensin 1-7 (pmol/mL)	~90	~180	[14]

*Measured with a microassay based on angiotensin I trapping by antibody.
